# “Wearing Two Hats Helped Me Look at the Bigger Picture”: An Interpretative Phenomenological Analysis of Being a Parent and a Clinician in Behavior Analysis

**DOI:** 10.1007/s40617-025-01077-6

**Published:** 2025-06-26

**Authors:** Victoria Burney, Clare M. McCann, Angela Arnold-Saritepe

**Affiliations:** School of Psychology, The University of Auckland, Auckland, New Zealand

**Keywords:** Parent engagement, Behavior analysis, Interpretative phenomenological analysis, Qualitative methods

## Abstract

Behavior analytic considerations of the role of parents in child-focused interventions are moving from a focus on parent adherence to concepts such as parent involvement, concordance, and collaboration. Despite this shift, little empirical work has canvassed the perspectives and experiences of behavior analysts or parents to support knowledge development in this important area of behavior analytic practice. The current study explored the experiences of two behavioral clinicians who are parents of children who have received behavioral intervention, with the aim of informing clinical work with parents. An interpretative phenomenological analysis (IPA) case study approach was applied, with interviews as the data collection method. Outcomes of the analysis highlight that for participants, the involvement of parents in behavior-analytic interventions relies on successfully navigating a continuum of expertise toward balancing whose knowledge holds more weight across the intervention context. Experiential themes generated in this study emphasize that clinicians who practice humility, listen actively, and value lived experience in establishing and delivering interventions will have the most success meaningfully involving parents in interventions for their children. Clinical implications of these findings are discussed, as well as future research directions.

Within behavior analysis, the active involvement of parents (broadly considered in this study to include biological and non-biological adults providing primary caregiving responsibilities for children) in interventions for their children is linked to better outcomes for parents (Kazdin & Whitley, 2003; Koegel et al., 1996) and children (Ingersoll et al., 2020; Najdowski & Gould, 2014; Nock & Ferriter, 2005). The field of behavior analysis has long recognized the importance of including parents in interventions to drive meaningful behavior change (Matson et al., 2009), to promote the maintenance of behavior change over time (Brookman-Frazee et al., 2009; Dogan et al., 2017), and to support generalization of outcomes to relevant environments when formal intervention ends (Moore & Symons, 2011).

In practice and research, this focus on involving parents and caregivers in interventions has historically been positioned as a concern with parent adherence, or fidelity, to treatment procedures (Patterson & Chamberlain, 1994). Following an initial call from Allen and Warzak (2000) to attend to variables impacting parent adherence to treatment protocols, subsequent behavior analytic considerations of parent fidelity in treatment have considered assessing child behavior as antecedent contributors to parent responses (Stocco & Thompson, 2015), and determining the function of parent nonadherence in order to select treatment approaches (Fryling, 2014; Hodges et al., 2020). Despite concerted efforts in the literature to identify, assess, and implement interventions to improve parent adherence, this continues to be an area of challenge for practitioners working with parents in child-focused behavioral interventions. Notably, despite a recognized need to identify variables relating to meaningful parent involvement in behavior analytic interventions (Pacia et al., 2023), scant behavioral research exists to specify those variables or conditions that parents and behavior analysts deem to be salient in this domain.

In response to the clinical issue of nonadherence or limits to the involvement of parents in intervention, more recent attention has been paid to broader conceptualizations of how parents are included in intervention, such as a focus on parent engagement (Burney et al., 2024a), collaboration (Marchese & Weiss, 2023), and concordance (Andrews et al., 2022; Pacia et al., 2023). Aligned with research on parent engagement in treatment within related allied health service provision contexts (see D’Arrigo et al., 2018; Klatte et al., 2024; Melvin et al., 2020; for reviews), the field of behavior analysis is beginning to investigate the issue of active parent involvement in behavioral interventions from the standpoint of compassionate care. Notably, drawing on a survey of parents involved in behavior analytic interventions, Taylor et al. (2018) argued for increased attention to and training in interpersonal skills for behavior analysts to develop compassionate practice aligned with parent preference. More recently, Marchese and Weiss (2023) suggested that promoting compassionate care skills in behavior analysis could both further the field’s efforts in social validity and create the conditions for parent collaboration within interventions. In this frame, emerging research on the topic of parent involvement within interventions is aligned with broader trends in the field of behavior analysis and may help to elucidate the relevant responses or behaviors clinicians can engage in, to address this specific clinical concern, and to strengthen the position of the field more globally (Melton et al., 2023; Rohrer & Weiss, 2023).

Notwithstanding moves from within behavior analysis toward relational and collaborative approaches to parent involvement, many facets of parent engagement remain under-researched. Importantly, little investigation to date has canvassed the perspectives of stakeholders (i.e., both parents and behavior analysts) directly to examine the factors that contribute to (or detract from) strong collaboration between parents and clinicians in the context of child intervention. Notable outliers include studies by Burney et al. (2024a, c), which used thematic analysis methods to explore understandings of parent engagement from the perspectives of behavioral clinicians and from the perspectives of parents accessing (or recently involved with) behavior analytic interventions. These studies offer insight into how each stakeholder group (parents and clinicians) understood parent involvement in interventions, noting the value of relational approaches in facilitating parent and clinician collaboration. While generating some insight into how parent engagement might be conceptualized, both studies were limited in scope (e.g., few participants in the geographically defined area of Aotearoa, New Zealand) and were homogenous in the methodological approach utilized for the investigation (Burney et al., 2024a, c). Further, parent engagement literature to date has not considered how parent involvement in behavior analytic interventions informs, or shapes, the responding of behavior analysts within their practice environments. This represents another interesting area of inquiry for behavior analysts interested in shaping their own behavior repertoires to achieve optimal parent and child outcomes (Taylor et al., 2018).

In the “fuzzy” (Critchfield & Reed, 2017) space of parent involvement in behavioral intervention, where the salient variables for change are relatively unknown, gathering insights from stakeholders with unique lived experiences of behavior analytic interventions, both from the perspective of delivering and receiving services, could offer behavior analysts data around what promotes, or detracts, from active parent engagement. These data may support refined definitions, measurements, and intervention approaches for behavior-analytic interventions that fully involve parents (King et al., 2022). Specifically, the perspectives of individuals with dual experience, that is, clinicians working in behavior-analytic intervention delivery who are also parents to children accessing behavior-analytic intervention, could provide a way to contextualize knowledge around parent engagement, which stimulates clinical practice. Such insights developed through qualitative research might offer direction to behavioral clinicians who do not have lived experience as parents but who hope to improve their skills in successfully promoting parent involvement within clinical work settings.

## Aims of the Study

This study aimed to explore parent engagement in behavioral interventions by canvassing the experiences from a unique perspective: that of practicing behavioral clinicians who are also parents of children who have accessed behavioral intervention. It was judged that analyzing the phenomenon of holding “two roles” or “dual hats” (i.e., having direct experience on both sides of the intervention context) would generate novel understandings of parent engagement in ways that were not available through qualitative analysis of data constructed with either clinicians or parents, holding lived experience of only one “role.” A case study approach using interpretative phenomenological analysis (IPA) was applied to develop insights that could inform the clinical practice behaviors of analysts toward fostering parent involvement in interventions.

### Research Questions

Aligned with the focus on gaining insight into what behavioral clinicians who are also parents experience at the intersection of these two roles, the overarching research question for the IPA study was:

*How does the experience of being a parent and a clinician in behavior analysis inform clinical practice?*

Further, theoretically derived questions were formulated during the conceptualization of the IPA study. These were considered secondary to the overall research question and were not explicitly presented in data collection activities (to preclude exerting undue influence over how participants shared their stories). Instead, secondary questions were used to guide the later stages of analysis (Smith et al., 2021). Questions included:

*How does being a parent to a child with additional needs give meaning to the participant’s clinical practice? What are the perceived benefits and drawbacks of having two distinct experiences for participants? What can clinicians learn/know in light of participant experiences on “both sides” of the clinical interaction? What do clinicians need to do differently to inform their own clinical practice with parents?*

A broad conceptualization of clinical practice was taken in the study to allow participants to share their experiences in ways that made sense to them without the constraint of specific terms or concepts (Smith & Osborn, 2004). This broad framing also had the effect of aligning the analysis with the intended audience (behavior-analytic clinicians with varying levels of experience) and with the overall focus of the project, toward stimulating change in the clinical practice of behavior analysts across work contexts. Although understanding parent engagement in behavior analytic interventions was a main driver of the study, we avoided the impulse to position the research as exploring “parent engagement” when engaging with participants, as it was possible to consider this as a clinician-driven construct, which may or may not account for the variety of ways being a clinician and being a parent could intersect.

## Methods

### Study Design

Interpretative phenomenological analysis (IPA) was selected as the analytic approach for the study. IPA is a qualitative research method that involves conducting a detailed exploration of an individual’s lived experiences, investigating how individuals derive meaning from such experiences (Smith, 1996; Smith & Osborn, 2004) to generate useful insights into particular events or contexts (Ashworth, 2008). IPA draws from three theoretical foundations or philosophical commitments. Namely, that of *Phenomenology* (the study of experience, specifically how that experience is available to and understood by the person with the experience; Becker, 1992), *Hermeneutics* (the way that meaning is ascribed by people to events, specifically a double hermeneutic, where researcher interpretations build on participants’ own interpretations of their experience; Smith, 2007), and *Idiography* (focus on the particular or specifics of individual cases as a means to generate insight; Smith et al., 1995).

The use of IPA was aligned with the overarching methodology of interpretive description (Thorne, 2016) in the study. Specifically, this analytic approach was thought to prioritize a phenomenological and idiosyncratic lens to explore the unique experiences of engagement in behavior analytic interventions by participants who had experiences on “both sides” of the clinical interaction. It was anticipated that findings from the IPA analysis would provide highly contextualized insights into the phenomenon of being a parent *and* clinician in ways that could further clinical understandings and, eventually, clinical practice (Shaw et al., 2014).

### Researcher Positionality

IPA was judged to be a particularly cogent methodological fit, given the positioning of researchers in this study as both insiders and outsiders to the topic area (Dwyer & Buckle, 2009). Specifically, the IPA approach acknowledges that holding “insider” status is not necessary to generate a credible study (Smith, 1996). Rather, ongoing reflection and interrogation of what the researchers *know* in relation to the analysis and stories of their participants is most important in developing the double hermeneutic layer and producing good quality research (Eatough & Smith, 2017). For the research team, who held clinical experience (the first and third author are Board Certified Behavior Analysts, and the second author is a speech-language therapist) alongside varying levels of personal experience (the first and second author were not parents of child/ren who had accessed behavioral interventions), this positioning as both knowledgeable and naïve in the phenomenon under study was accommodated within the IPA methodology.

A constructivist orientation to knowledge was held by the research team which informed decisions across all stages of the study. This constructivist epistemology, or orientation, assumes that all knowledge is situated in an individual’s context (e.g., their learning history, their cultural, physical and social environment) and is created, or negotiated, through language (Madill et al., 2000). This positionality assumes that there is no one right way of understanding phenomena under study but rather suggests there are “multiple interpretations to be made of any phenomenon, which depend upon the position of the researcher and the context of the research” (King, 2004, p. 256). This way of thinking informed decisions around which analytic methods to adopt, the selection of participants, how data collection opportunities were structured and conducted, and how outcomes of the study were described in the write-up.

### Ethics Statement

The study was granted ethics approval by the University of Auckland Human Participants Ethics Committee through an amendment to an existing application (Reference Number: 23280) until 02 December 2025. Pseudonyms were self-selected by participants and are used throughout to protect confidentiality. Some demographic information is excluded from the write-up to further promote the confidentiality of participants.

### Participant Selection and Sample

This IPA study was generated as part of a broader program of research, which applied various qualitative approaches to exploring the perspectives and experiences of parents (*n* = 15) and clinicians (*n* = 13) in behavior-analytic interventions for children (see Burney et al., 2024a, c for publications relating to this project). Of participants involved in the larger project, two individuals self-identified as behavioral clinicians who are also parents to children who have received behavior analytic intervention, representing a unique third group within the research. No other participants or individuals involved in recruitment for the broader project tacted having “dual” experiences of being both a parent to a child accessing ABA intervention and a clinician who works in behavior analytic intervention delivery. It was acknowledged that these parents, who are also practicing clinicians, likely have insight into the practice of behavior analysis in Aotearoa, which is unavailable to other participants. Given the small population of Aotearoa, New Zealand, and the small size of the behavior-analytic community, it was thought that a case study approach investigating the experiences of these two participants would be best aligned with the pragmatic and epistemological positioning of the current study, as well as the aim of the study to inform understandings of how behavior analysts can promote parent involvement. For these reasons, a case study approach was chosen, which utilized an existing group of participants (over seeking a larger sample) to allow for a comprehensive analysis and necessary resources to develop an interpretative “dialogue” (Smith et al., 2021, p. 47) in the study. In this decision, the informational power of the data gathered was prioritized (Malterud, 2001; Malterud et al., 2016) over approaches such as representativeness or saturation (Braun & Clarke, 2024a, b), which were deemed to be a less coherent fit with the constructivist perspective of the study.

### Participants and Recruitment

Both parents who had taken part in data collection activities during a previous phase of the project (Burney et al., 2024c) agreed to participate in the IPA study, including consenting to further analysis of initial data products and taking part in a subsequent interview.

Participants self-identified as behavior-analytic clinicians who were also parents of child/ren who had received behavior-analytic interventions. Participants described themselves as having lived experience in behavior analytic interventions informed by both personal (parenting) contexts and professional (clinical) contexts. Participants used the self-selected labels “dual hats” and “dual roles” to describe this phenomenon. Both participants were mothers of children with developmental delay and/or neurodivergence who worked as behavioral practitioners at the time of the study. Mia was a behavior analyst with more than 8 years of clinical experience implementing behavior-analytic interventions across populations (including developmental and intellectual disability, brain injury, and autism). Mia became a parent to a daughter (8 years old at the time of the study) who accessed behavior analytic intervention during her training in behavior analysis. Anna was a behavioral clinician who retrained in behavioral approaches after becoming a parent to a son with developmental delay, autism, and behaviors of concern (14 years old at the time of the study). Anna had more than 10 years of experience working clinically in behavior change. Participants were living and working in different regions of Aotearoa, New Zealand, at the time of the study for separate non-governmental organizations providing behavior analytic services. Both had experiences with accessing behavioral interventions for their children (in the form of home-based intervention) within the two years prior to taking part in the study. Despite being part of a small pool of behavioral clinicians in Aotearoa, New Zealand, the participants in the study did not hold any professional (or child-focused intervention) connections with the first author at the time of the study.

### Data Collection

Data in this study were drawn from two interviews with each participant, conducted approximately a year apart. Data from an initial interview (carried out during an earlier phase of the project; Burney et al., 2024c) was supplemented by a second interview with each participant to allow for the gathering of in-depth information aligned with the specific focus of the IPA analysis. Interviews centered around exploring the intersection of parent and clinician roles for each participant, aligned with the research question guiding the analysis. Consistent with the perspective that qualitative interviewing involves “a conversation with a purpose” (Smith et al., 2021, p. 54), no interview schedule was employed during the second interview. Few researcher-driven questions were posed to participants during the second interview, with more time provided for each participant to share their stories with minimal researcher interjection.

Existing data generated during an initial interview with each participant was incorporated into the analysis at the stage of data familiarity (see *data analysis* below). Despite being constructed under a different set of analytic assumptions and with the use of a semi-structured interview schedule, interview transcripts generated from the first interviews explored how participants made sense of their parenting and clinician experiences to some degree and so were judged to be important for informing the IPA analysis. Questions posed during initial interviews focused on parent descriptions of their involvement with behavior analytic interventions, experiences of working with clinicians, and perspectives of aspects that promoted or detracted from engagement (for additional details on the interview schedule, see Burney et al., 2024c).

All four interviews were led by the first author and lasted approximately an hour for each participant across data collection opportunities (range 51–64 min). Although relatively equal in length, second interviews moved more quickly into the sharing of accounts and experiences, with less time spent building rapport. Several factors are thought to have contributed to this shift, notably that the participants and researcher were known to each other in the second interview and had experience in co-constructing an interview context (Olson & Brinkmann, 2011), and that the interviewer had honed skills in facilitating research interviews, through practices of reflection and peer debriefing (Yardley, 2015).

Individual interviews were held in person (Mia) or using remote meeting technology (Zoom; Anna) based on participant preference and availability. Interviews were audio recorded using either an Olympus DS-2 digital voice recorder or Zoom audio recording software, depending on the interview modality. Interviews were then transcribed by the first author using a consistent “intelligent verbatim” approach (McMullin, 2023). Participants were offered an opportunity to read and revise any elements of their transcripts; however, no changes were requested by participants.

### Data Analysis

An IPA case study approach was employed to analyze the data. Initially, there was disagreement within the research team around the conceptualization of “cases” in this analysis. This confusion is echoed within IPA literature, which acknowledges variability in how researchers define a “case” and what data products are expected to formulate a case (Smith & Osborn, 2015). Ultimately, this was resolved through the stages of analysis, where it became clear that the distinction of “cases” was somewhat arbitrary, as one participant’s experience could not be effectively analyzed without combining both data products. Although participants varied in their experiences of being both clinicians and parents (notably in whether they were a parent or a clinician first), it was thought that there was enough similarity in the experience of these participants to generate useful insights about what it meant to the participants to experience both being parents and clinicians, while simultaneously allowing for “micro-analysis” (Smith et al., 2021, p. 46) of the contrasts and tensions present between participant accounts of this phenomenon.

Steps for conducting an IPA study, described by Smith et al. (2021), were followed. Specific details for how each step was achieved in the current study are noted below. A team approach to data analysis was utilized in the study, with the first author leading the first three analytic steps alongside regularly scheduled discussions with the second and third authors. Steps four to seven for both participants were completed with the whole team during frequent (approximately fortnightly) data analysis workshops.

### Step 1: Reading and Re-Reading

This step involved revising written transcripts (from the initial and subsequent interview for the first participant) while listening to audio recordings of interviews numerous times. This process provided the first opportunity for “immersion” in the data products (Smith et al., 2021, p. 78), allowing for awareness of what was discussed, the flow of the conversation, and specific contextual cues salient in the interviews. The written transcripts were reviewed at least twice, with the intention to read “cleanly” without adding annotations, notes, or comments. This proved difficult, as reading the transcripts with an eye to the research question led the first author to want to make notes and underline ideas that felt important.

### Step 2: Exploratory Noting

Here, steps one and two bled together. Initial notes and underlined sections were added across each transcript in its entirety. Repeated readings resulted in more exploratory notes being added to the right-hand margin of the printed transcripts. Some notes consisted of verbatim quotations or identification of specific language used by the participant; others paraphrased a key idea or message the participant shared (Eatough & Smith, 2017). Notes included the identification of similar messages across the transcripts or areas of disagreement within each interview. Some interrogative questions were also generated in the form of “If x is like y, what does that mean for z?”. Researcher reflections developed from interactions with the transcripts, but not directly “tied” to the data product, were journaled separately (Smith & Osborn, 2015). This step enabled critical reflection on the process, the analytic direction, and potential researcher-driven biases as the analysis developed.

### Step 3: Constructing Experiential Statements

This step involved re-reading each transcript, paying closer attention to the exploratory notes, in an effort to generate experiential statements that summarized or took account of the exploratory notes for each part of the transcript. Experiential statements were added to the left-hand column of the transcripts. This stage embodied the double hermeneutic characteristic of IPA, with researchers “trying to make sense of the participant trying to make sense of what is happening to them” by applying analytic approaches “self-consciously and systematically” (Smith et al., 2021, p. 3). This step involved a tension between reading the transcripts (data generated by the participant) and reading exploratory notes (researcher interpretations of the data) and attempts to balance commitment to the idiographic details of the participant’s account with initial attempts at analysis.

### Step 4: Searching for Connections Across Experiential Statements

In efforts to facilitate group analysis, handwritten experiential statements were compiled into electronic tables. This allowed experiential statements to be easily shared with the research team, copied, cut out and moved around without compromising handmade notes on the transcripts (master copies). Group analysis workshops were scheduled every two weeks to allow for close connection with the data products while providing space to debrief, reflect, and build awareness of potential biases impacting the analytic process (including researcher positionality, assumptions about the process of analysis and expected findings, and tensions around the ultimate audience for outcomes and the function, or use, of the analysis in dissemination; Charlick et al., 2016; Shaw et al., 2014).

Steps 1–4 were completed for data generated by the first participant. Once this was complete, all four steps were then initiated for the second participant. While Smith et al. (2021) suggest keeping cases separate until step 6, in this analysis, comparisons across cases started to form part of the discussion at step 5.

### Step 5: Naming the Personal Experiential Themes (PETS) and Consolidating them in a Table; Step 6: Continuing the Individual Analysis of Other Cases

Although progression through analytic steps is presented as a linear process, this stepwise approach was difficult to honor in practice. The research team attempted to “park” data and reflections from interactions with the second participant while naively, or inductively, exploring and analyzing data products from the first. However, this was problematized by the timing of data collection opportunities, which were scheduled concurrently with analysis owing to participant availability. Further, the transcription of the second interview by the first author occurred during the initial stages of analysis, making it challenging to separate insights across participants. In this stage, secondary research questions were used to interrogate the data and to refine or adjust thematic structures that were developing across cases as part of regular research team meetings.

### Step 7: Working with Personal Experiential Themes (PETS) to Develop Group Experiential Themes Across Cases

**Fig. 1:**
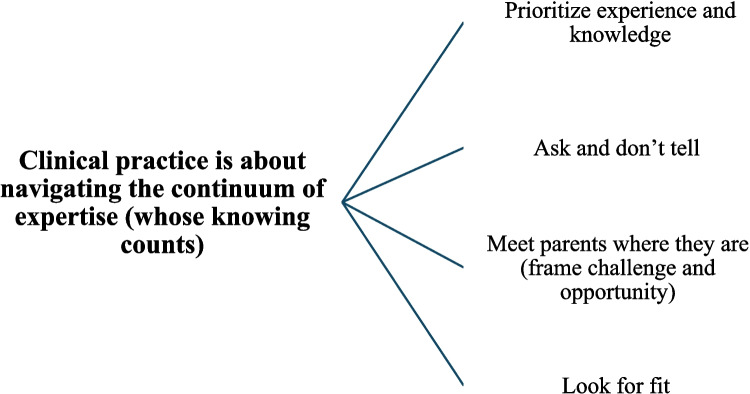
Group experiential theme and subthemes developed within IPA analysis

During this step, ideas around candidate experiential themes were revisited, and experiential themes were reconsidered, including their relationship to each other (e.g., their hierarchical arrangement). This step was primarily achieved by comparing visual representations of working thematic structures with each participant’s experiential statements and with consideration of the research question and aim of the study. Through interrogation of experiential themes that were most salient and impactful from the perspective of participants and using the interpretative lens of the research team, a final thematic structure was developed (Fig. 1).

#### Rigor

Various steps were taken to support the rigor and integrity of the research process, in line with a commitment to coherence across the epistemological assumptions, methods, analysis, and presentation of outcomes in the study (Braun & Clarke, 2024b; Levitt et al., 2018). Strategies including: regular journaling and critical self-reflection on the part of the first author (Finlay, 2002), opportunities for group discussion and peer debriefing (Spall, 1998) with researchers both within and outside the study, and the development of an audit trail of decisions and products throughout the study (Carcary, 2009, 2020), were utilized to bolster the rigor of the research.

## Results

Figure 1 shows the group experiential theme and subthemes generated within this analysis across both case studies. Throughout this section, participant quotes are provided to support ideas generated in the analysis. Some quotes have been adjusted from original comments in the transcripts for brevity, signified by ellipses.

### Clinical Practice is About Navigating the Continuum of Expertise

The group experiential theme developed in this analysis is that of successfully navigating the continuum of expertise. For both participants, navigating the continuum of ways of knowing (or holding expertise) within the intervention context was described as the central work of behavioral clinicians toward fostering interventions that fully involve parents. For participants, this continuum of expertise is present and pervasive in every clinical intervention context; that is, a part of all clinical interactions. However, having rich experiences on both sides of the expertise continuum helped to “bring into focus” or make evident this process of navigation, in ways that might be less visible or available to clinicians who do not hold dual roles as a clinician and a parent. For Anna and Mia, wearing two hats provided an opportunity to stand back and acknowledge the tensions that exist between different ways of knowing in a child-focused intervention context and how these can be successfully balanced throughout an intervention.

Within this global theme, focus on successfully navigating between both types of expertise (or ways of knowing) was described as a way to help parents feel engaged and connected and ensure they get what they need from interventions, while supporting clinicians to feel they are providing useful input to the families they are serving. The tensions and challenges of navigating this continuum of experience were borne out in a range of examples through participant recounting of experience. These include the subthemes: *prioritize experience and knowledge, ask and don’t tell, meet parents where they are, and look for fit.*

In this analysis, these subthemes present contexts/domains in which parents and clinicians navigate who holds power and agency within a collaborative child-focused intervention context.

### Prioritize Experience and Knowledge

Fundamentally, for participants in this study, successfully navigating tensions within an intervention context starts with seeing (and valuing) the knowledge, experiences, ideas, and expertise parents bring to interventions. Necessarily, this requires shifting preoccupation with, or de-prioritizing, the experiences *clinicians* bring to a child-focused intervention. The process of navigating expertise was exemplified in the exploration of how much (and with what level of genuineness) parents’ lived experience informed the development of an intervention, over clinical judgment or experience. For Anna, each parent’s experience with their own child immediately positions them as experts in the intervention:

It’s about seeing that the parents have the most experience. I see a lot of clinicians who go in thinking they know everything, and it’s like, unless you’re a lived clinician then I feel like you can’t know … it’s about listening to those parents as experts. (Anna, Interview 1)

Mia described a need to step back and consider the whole picture, including where parent expertise lies, rather than leading with your own clinical ideas of what works:

Wearing two hats helped me to look at the bigger picture, not so much of ... what skills this child is lacking or what strategies need to be done to teach this skill, more … taking steps back and looking at the context: is this family well supported? Are they burned out? Do they have services in place to help them with respite? Are they sleeping? Everything, just checking everything. (Mia, Interview 2)

On the basis of their experiences holding dual roles in behavioral intervention, participants both explored the place for awareness (or paying attention) on the part of clinicians to better understand the relative expertise of all parties and more carefully navigate whose knowing is prioritized at each stage of an intervention. When clinicians come to identify their potential biases and assumptions around whose knowledge counts, they can deprioritize the specific clinical concerns and instead ask, “What can I offer these parents?” challenging inherent power dynamics.

Rather than coming into work with parents “knowing” what intervention was required, Mia described that “I would bring in ideas, offer some input, take perspective, step back” (Mia, Interview 1). In this way, prioritizing parent knowledge shifts the boundaries around interventions in ways that “push parent voice to the top” (Anna, Interview 2) and provide opportunities for parents to self-advocate, for example, by saying, “Sorry I disagree with you.” (Mia, Interview 1). For Anna, this prioritizing of parent experiences and knowledge manifests in giving parents space to say what they know and don’t know (or want to know):

If they want education, we will always talk about that. Or if they want, we talk a lot with wider family, because often it’s getting other family acceptance. So, I guess I’m always coming from that lens … we need families to be resilient enough to care for their own people, with the very limited funding and supports that they get. One way to do that is to make things as easy as possible in that family, to hear what they need. (Anna, Interview 2)

For both Anna and Mia, negotiation around whose experience “counts” involves a level of humility on the part of clinicians to offer parents what they need rather than, perhaps, what seems clinically indicated in order to “give them the confidence to keep going” (Mia, Interview 2). Prioritizing parent expertise, in this account, involves giving away (or negating) some of the inherent power held by clinicians to enable parents to determine change. Mia offers this perspective:

When you go to a family’s home and you over-focus on the challenges of the children, you make the parents feel even worse about themselves. Then they’re likely to avoid you or avoid the service. Because you are actually making them feel completely powerless … [Clinicians] will say that they’re disengaged but they are not empowering the parents to say what they need. (Mia, Interview 2)

### Ask and Don’t Tell

Navigating the continuum of whose expertise matters is especially critical at the outset of intervention, when clinicians are attempting to build therapeutic relationships with parents to establish their involvement. Consistently, participants highlighted the need to “ask and don’t tell” with parents to build a working relationship and exemplify that parent knowledge holds capital in determining the intervention context.

For both participants, this begins with simply creating the conditions for listening. Anna reflected on the space of listening in being a “genuine human” and not pushing your expertise to the top.

It’s about listening to those parents. That would be my biggest advice: that clinicians really need to listen. I’m sure a lot of them go in with preconceived ideas … you can form a picture and it’s that bias. I like to go into that first meeting without really knowing anything and then just … hearing what they say. (Anna, Interview 1)

Additionally, Mia framed this process of developing connection as one of giving parents more time and space to share their context and not “rushing”: “Understanding things that are important to the family, before starting hands-on work with the child, it will make the service so much more effective” (Mia, Interview 1). Where this can fall down is when clinicians, motivated by other competing variables, rush the process of listening and building connection: “They [clinicians] come in thinking too much of the problem and how to fix it, instead of holding space for parents, to understand where they are coming from” (Mia, Interview 2). Anna noted that often, clinicians are in such a hurry to get things going they lean on assumptions as a shortcut to understanding a family’s context, rather than asking parents directly:

Clinicians need to wait until the assessment is done before they start making those assumptions. Because they come in immediately, and they look around the house, and they think, ‘Oh yeah, this is- this is what’s going on here.’ Everyone’s trying to look for that- they’re trying to sort out their assessment before they’ve actually fully done it. And I think that’s ... detrimental. It doesn’t honor parents. (Anna, Interview 2)

Participants both explored the necessity of suspending judgment based on what you read, are told, referral details, or first impressions. Building on their experience, participants shared that judgment (or closed-mindedness) can move the intervention toward one that legitimates clinical experience over the parent’s lived experience and limits the parent’s ability to acknowledge their understanding of their child and their child’s needs. By asking (rather than telling), clinicians can let parents dictate the narrative. Allowing parents to communicate their understandings and expertise without framing within a clinical lens avoids feelings of judgment on the part of parents by clinicians. Anna comments that “just being able to talk about the behavior, and also with no judgment” (Interview 1) helps to navigate the continuum of expertise by giving parents space to share. Further, Anna describes how clinicians need to be “thinking about which questions they are asking. When you start asking those questions about parenting, or, you know, parent characteristics, things ... that can be where we lose them, because families feel judged, straight away.” (Anna, Interview 2).

For both participants, “telling rather than asking” holds an inherent risk for eroding parent collaboration within interventions. Mia summarized the idea that telling parents what to do does not help in creating a collaborative intervention context, as it risks damaging trust:

Imagine these parents, they are already feeling like they’re failing their child... and I come in and tell them what they’re supposed to do? No. They know what they are supposed to do. But they’re not well enough to make any changes. They’re exhausted. And so, I don’t feel like it’s my place to go there and make them feel even worse about themselves. (Mia, Interview 2)

Instead, offering support rather than specific intervention tools or strategies at the outset is protective (“more of... giving them support and letting them know I understood” Mia, Interview 2). For Anna, this means “I’m giving advice to families, rather than telling them what to do … cause I don’t think that ever works” (Anna, Interview 2).

Mia reflected that in her experience, the process of asking parents, rather than telling them, involves a level of perspective taking on the part of the clinician:

A lot of clinicians lack perspective-taking. They don’t get the parent’s perspective. If the clinicians put time aside to build that rapport with the parents, to understand where they’re coming from and where they get stuck, if they shift the parent’s behavior, the parents will make changes for the child. (Mia, Interview 2)

Mia described this process as the building of trust, where over time and through repeated pairing, clinicians come to be viewed by parents as trustworthy and as holding genuine care for their child and their family, purely through prioritizing listening and nonjudgmental communication. In this way, asking parents (rather than telling parents) helps clinicians navigate the continuum of expertise such that strong, trusting relationships are developed within intervention contexts to support meaningful and ongoing parent involvement.

### Meet Parents Where They Are (Frame Challenge and Opportunity)

Within child-focused behavior analytic interventions, navigating expertise—or whose way of knowing (e.g., identifying contingencies) carries more weight—is exemplified in the tensions between acknowledging the inherent challenges of parenting children with additional support needs and framing these challenges toward a more productive outlook. In this analysis, meeting parents where they are involves flexibility and humility on the part of the clinician to create a safe space for parents to express the difficulties inherent in their lived experience, while sensitively creating opportunities to see the potential and value of interventions for their child and family.

Participants described awareness and keen understanding of how challenging it can be for parents who are seeking supports for their child/ren, stemming from their own parenting experiences. For Anna, the experience of parenting a child with additional support needs is a “tough” one, framed as difficult or “hard”: “It’s hard, it’s demoralizing, you’ll hear that time and again from parents… you are fighting and its hard” (Anna, Interview 1). Anna explains that she sees parents as always in survival mode or in spaces where “the mental energy has gone on just coping” (Interview 2), informing parent perspectives and positioning interventions in the space of difficulty or challenge. For Mia, this experience is one of constant pressures and demands:

“You really feel lonely, you get frustrated, you feel like no one understands you. Eventually you just stop sharing and you isolate yourself” (Mia, Interview 1).

In the context of seeking support, the parent’s experience of the challenge is further exemplified:

“Having to run around and attend appointments, managing all that and having to chase professionals, doctors, support needs services, schedule therapy, it is non-stop! It is hard” (Mia, Interview 1).

In the realm of clinical work, both Mia and Anna describe the role a clinician can play in recognizing where parents are at in understanding or processing their unique situation while sensitively navigating between providing time for parents to express their feelings and creating opportunities for parents to view situations from a more optimistic lens. In explaining this balance, Mia described that parents “have to be in the right frame of mind” (Interview 2) in order to hold the emotional and practical challenges of their parenting journey together with the belief that things can improve and that there is benefit or “real hope” (Interview 2) in engaging with child-focused interventions. Anna explained that sharing her experience was a way to offer hope to some families for progress and change by recounting how she had lived through the struggle and grief:

Lived experience … is being able to give hope to parents that, yes, it is hard, but it changes. When parents can see that you’ve come out the other side, you know? Ok, he’s 14, we’ve managed this long, nobody’s died, that’s a good thing! That’s always reassuring, isn’t it? (Interview 1)

By contextualizing her own situation and how her thinking has changed over the years of parenting her son, Anna is able to offer this insight to families, to identify where they are positioned in understanding their situation, and to help them frame their experience in a way that balances difficulty with the potential for change afforded by intervention and supports. Similarly, for Mia, experiences as a parent allow her to share her learning with other parents in a way that validates what they are going through while providing an opportunity for optimism in intervention:

I [am] able to give them words of comfort, to help them process their grief and what they need to get through. The first thing I do is definitely acknowledge how tough it is. And if they give me any space to say anything I would suggest if they were open to ABA, I would also recommend that, so the conversation would really... be dependent on how open this parent was. (Mia, Interview 1)

Participants in this analysis caution that finding the balance between providing space for parents to express the challenge of their situation and prompting other ways of thinking is difficult. For Mia and Anna, this requires careful reflection on the part of the clinician to gauge where a family is positioned and decide the relative value of reframing this experience for involving parents in interventions and not “turning them off.” Anna describes this tension:

I will tailor what I say to a family and their individual circumstances. Definitely. There are some families who are still very much in the grief stage and don’t … want to hear about the positive sides. They do just want to focus on what’s going wrong, how horrible it is. And so, it’s ok to let them stay in that space for a while because the child they thought they were having and what they have is … sometimes very different. And it is hard for families, and they all grieve at different rates. But I’ve always seen it as a ‘Ok, this is what we’ve got. What do we do next?’ kind of thing. (Anna, Interview 2)

### Look for Fit

For participants in this analysis, with experience on both sides of the expertise continuum, a key area where negotiation takes place is in positioning the intervention to fit the family context (while also aligning with the service delivery context). In this subtheme, tensions exist around clinicians letting go of power to decide and instead giving control to parents to imagine an intervention that matches their needs and resources.

Mia explains that this process begins by developing a fuller understanding of the learning histories and environmental variables relevant to each family and finding out what is possible for that family to achieve.

I usually sit down with the parents and I do a, a thorough check. I just, I ask. I ask: do you get respite? What services do you have in place? And then I pick up on what they need. And I let people know, that this family needs this and that. I’m happy to provide strategies, but families won’t be able to implement until they have those other things in place. So, I usually advocate for that first. (Mia, Interview 2)

For Anna, who was a parent before practicing behavioral interventions, acknowledging the parent’s limits and capacity (e.g., available resources) for doing so is essential to shaping what intervention is offered.

Actually, people don’t understand how hard the job is and 24/7 supervision is incredibly taxing. So, being able to just make little changes. Like, I’m not the clinician who goes in and writes a 10-page plan with ridiculous goals and strategies. Often, my goals look like, ‘We’re going to put in positive strategies that will implement quality of life for the person and their family.’ That’s generally it, and then we will put in- even using a first and then. Or helping with toilet training. Because if you could help with, just say toileting, and getting them so that they’re not doing washing every single day or spending hundreds of dollars on pull-ups and things, usually you find that other behaviors will decrease because you have put in some positive stuff. Small stuff.” (Anna, Interview 2)

Critically, recognizing variability in service delivery models and taking time to understand parent expectations around particular services is a way to avoid setting unrealistic or unfounded expectations and, ultimately, to make sure the intervention being offered is a practical fit for that family. For both participants, the breakdown in parent involvement and collaboration comes when clinicians “really push families” (Anna, Interview 1) and are inflexible in their expectations or delivery, leading parents to a place where: “they know what they are supposed to do. But they’re not well enough to make changes. They’re exhausted” (Mia, Interview 2). In this analysis, such inflexibility—or prioritizing clinical fit over parent and family fit—often means families pull away or refuse services, even if they want and need the support. Mia explains: “Parents push them away, it’s too hard, it’s too much. Too much pressure. The demand is too much. And I feel like a lot of clinicians don’t see that. They miss that point.” (Mia, Interview 2). Anna recounts that sometimes the way interventions are structured or delivered puts families in a space where: “parents just couldn’t … they don’t have capacity to do that” (Anna, Interview 2). For participants in this analysis, successful behavior analysis is about balancing the clinician’s impulse to know and decide with awareness of where families are at and what they can do: “I can see what I think would make a difference, but parents just don’t have capacity to do it. So, what’s the value?” (Anna, Interview 2).

Participants explored that sometimes, it is not about the intervention specifics, as much as getting the timing right and making space, for now to be “not the right time.” Anna explains:

When parents say to me, ‘Look, this is just not the right time,’ … that is exactly one of our options when we early terminate, not the right time. And that’s ok. Because if there’s huge family pressure and things happening … that will be contributing to behavior. But you’re still got to deal with that before you can deal with the behavior. And sometimes that, that’s the hardest thing [for parents], is realizing—and saying—that actually ‘now isn’t the right time.’ (Anna, Interview 1)

In order for clinicians to fully involve parents, sometimes they need to step back and consider if the personal resources available to families “match” what clinicians are offering or if they need to change what is offered. Mia describes awareness of parent positionality as determining how intense interventions can be: “I can’t expect a burnt-out mum to make changes when she can’t look after her own health. They have to be in the right frame of mind to implement anything” (Mia, Interview 2). Often, this means giving parents the agency and relative power or control over the situation to “choose to say yes” or to “choose to say no,” without the risk of being labeled as noncompliant or disengaged by clinicians (or necessarily contracting contingencies of punishment associated with discharge from services). For Anna, this is about supporting parents to develop their own agency to decide and to negotiate what is being offered in service of “helping” that child and family:

And it’s like, but... what if they can’t? Like that’s … it’s not that they won’t, it’s that they can’t. Because what you’ve decided to do, all the homework or the data collection that you want them to take, all of this is actually just not possible at the moment. So, what can you do to make that easier? (Anna, Interview 2)

Importantly, being flexible and responsive to parents’ needs and wants while recognizing that parents “can’t disengage from their child” (Mia, Interview 2) and can only choose to be more or less involved in each intervention setting will support clinicians to offer interventions that are a good fit for parents, promoting active involvement.

## Discussion

This study aimed to explore parent involvement in behavioral interventions from a qualitative lens, using IPA to analyze the experiences of two participants who are behavior clinicians and parents of children who have received behavioral intervention. Aligned with the objective to further behavior analytic practice, the study aimed to develop insight into the specific behaviors that analysts can engage in to address the important, yet complex space of parent involvement within child-focused behavioral interventions.

The key experiential theme of the analysis—that successful collaboration with parents involves navigating the expertise parents and clinicians hold within an intervention environment—offers insight into the interaction of parents and behavior analysts not captured in previous studies. Specifically, for participants in this analysis, there are implicit tensions coming into an intervention with parents over whose way of “knowing” and experience is prioritized and who holds power to shape or influence how the intervention proceeds. Informed by their experiences on both sides of the clinical interaction, participants in this study highlighted that clinicians who can balance parents’ knowledge and insight alongside their own clinical objectives are more likely to create successful conditions for active and sustained parent involvement in interventions for their children. Approaches such as suspending judgment, listening carefully to parents (or asking rather than telling), identifying where parents sit in processing their unique circumstances and affording parents an opportunity to frame their perspectives on interventions all contribute to building a collaborative relationship that promotes the involvement of both parties. Necessarily, from the perspective of participants in this study, clinicians are charged with bringing parents into interventions on an even footing and managing dynamics of power, choice, and control, to allow parents to advocate for their child in ways they prefer. Clinicians, then, need to avoid valuing their own clinical expertise above other ways of knowing, instead prioritizing parent knowledge in developing a good fit intervention.

Although these findings are novel, they hold congruence with research on parent collaboration in child treatments, including research generated within other allied health fields (e.g., Burney et al., 2024b; Klatte et al., 2024). Importantly, these outcomes position parent involvement as a dynamic process built on alliance and relational responding, a finding echoed in studies exploring parent engagement in speech-language therapy (Melvin et al., 2021), occupational therapy (D’Arrigo et al., 2020), and rehabilitative health treatments (Phoenix et al., 2020a, b). Aligned with programmatic parent engagement research in other disciplines (King et al., 2022), the outcomes of this study reiterate that attending first to building connection and establishing the value of lived experience creates the conditions for successful and lasting involvement of parents in therapeutic inputs for their children. Primarily, this process begins with open, genuine communication and willingness to ask for insight rather than “tell” or give parents a clinically informed answer.

When considering behavioral conceptualizations of parent involvement, the outcomes of this study support the conclusion that behavior analysts need to apply relational, compassionate approaches to working with parents rather than focusing on parent behavior in the form of adherence or fidelity. This finding aligns with Burney et al. (2024c), where parents expressed that the specific components of a behavioral intervention (i.e., the technical elements of the treatment package) were less critical in determining how involved parents were in the intervention than other variables such as how well the clinician identified parent needs and concerns when designing interventions. More broadly, the findings of this study support a behavior-analytic focus on compassionate care, as it relates to building working relationships with stakeholders toward more robust clinical outcomes and bolstered acceptability of behavior-analytic interventions (Rohrer et al., 2021; Rohrer & Weiss, 2023; Taylor et al., 2018). As highlighted in the conceptual work of Melton et al. (2023), these findings offer support to the general call for behavior analysts in clinical practice to develop their interpersonal skills (or soft skills) toward building better working relationships with parents before technical skills are emphasized. This outcome lends weight to recent emphasis in the field of behavior analysis on training analysts to master the skills of practicing in compassionate and relationally informed ways (LeBlanc et al., 2020; Nohelty et al., 2024).

Notably, while many ways to promote parent involvement were explored in this study, participants explained that from a parent’s perspective, the label “engagement” may not be a meaningful way of conceptualizing parents’ position in interventions. Indeed, it seems that parents, as described in this analysis, do not have the luxury of disengaging from their children or the support needs of their children. In this way, the term engagement may be better understood as a clinical construct, which helps clinicians to tact parent behaviors that connote connection and collaboration (e.g., effective communication, attendance, sharing of ideas) and behaviors that suggest limited involvement (e.g., missing appointments, lack of communication, not offering feedback). From this analysis, we could consider that labels such as parent collaboration or parent involvement better describe parent experience within behavior analytic intervention, while “engagement” and “disengagement” are reserved as clinical discriminations, mostly useful for practitioners.

Although the case study nature of this research (and the idiographic and phenomenological focus of IPA) precludes broad generalization of findings, the outcomes of the study go some way to informing the clinical practice of behavior analysts working with parents on child-focused interventions. Importantly, findings suggest that honing interpersonal skills around active listening and empathetic relating to parents will help to address challenges by successfully involving parents. Further, findings emphasize that behavior analysts in clinical practice need to develop skills and fluency in compassionate care approaches to best support their clients (Marchese & Weiss, 2023; Nohelty et al., 2024). This underscores the importance of conceptualizing relationship as a necessary antecedent condition for success and considering parent adherence—if at all—as a product or consequence of this antecedent manipulation. Compassionate care approaches align with a shift from focusing on parent fidelity with treatment to prioritizing parent expertise in assessing, developing, and delivering interventions that are a good fit for parents, in service of actively involving parents in interventions for their children.

This study applied a qualitative methodology (IPA), relatively underutilized in behavior analytic research, to investigate parent involvement. In this example, IPA offered an approach to research that prioritized participants’ recounting of individual experiences toward making measured generalizations of perspectives to behavior analytic practice and informing behavior analysts in their work with families. The capacity for findings developed under qualitative assumptions to answer practical questions and to inform clinical behaviors is bolstered by the use of IPA, and specifically the attention given to the idiographic, phenomenological accounts of participants in ways that can be viewed through a lens of behavior analytic philosophy (Burney et al., 2023).

### Future Directions

This case study offers a small but important step toward conceptualizing the place of parents in behavior-analytic interventions. There remains ample scope for further investigation into the phenomenon of parent involvement from a behavior analytic account. Future studies may wish to use qualitative approaches to investigate the experiences of wider groups of stakeholders, to include greater numbers of participants with diverse experiences with behavior analysis, and to consider collaboration with other fields pursuing parent engagement research alongside parents, using participatory research approaches. Gaining further insight into the phenomenon of parent engagement may move the field of behavior analysis closer to conceptualizing, defining, measuring, and promoting the involvement of parents in ways we are not readily able to do currently. Exploring the role of cultural and social contingencies in influencing perspectives and experiences of both clinicians and parents (as these pertain to involvement with behavior-analytic interventions) is a line of research that may support the generalizability of the current findings and identify other areas in need of further empirical investigation.

## Conclusion

By sensitively navigating the continuum of knowledge or expertise and providing parents with the agency to speak, to be heard, and to exert influence over interventions for their children, clinicians can foster genuine involvement of parents in child-focused behavior analytic interventions. The interpersonal nature of this finding supports the contention that behavior analysts need to be trained in—and fluent in applying—compassionate care approaches in interactions with parents to further this aim of collaboration. Current research into parent engagement in behavioral interventions supports broader trends in the field toward interrogating the social validity and acceptability of behavior analytic input for consumers and stakeholders (such as parents) and toward positioning behavior analysis as a more compassionate and responsive science of behavior change. Further research will help to develop knowledge around parent engagement and more concretely inform the clinical practice of behavior analysts working with parents.

## Data Availability

Due to the nature of the study and the context in which data were gathered, the dataset of this study is not publicly available to preserve participant confidentiality.
